# Biscuits with No Added Sugar Containing Stevia, Coffee Fibre and Fructooligosaccharides Modifies α-Glucosidase Activity and the Release of GLP-1 from HuTu-80 Cells and Serotonin from Caco-2 Cells after In Vitro Digestion

**DOI:** 10.3390/nu9070694

**Published:** 2017-07-04

**Authors:** Nuria Martinez-Saez, Christina Maria Hochkogler, Veronika Somoza, Maria Dolores del Castillo

**Affiliations:** 1 Food Bioscience Group, Department of Bioactivity and Food Analysis, Institute of Food Science Research (CIAL, UAM-CSIC), C/ Nicolás Cabrera, 9, Campus de Cantoblanco, Universidad Autónoma de Madrid, 28049 Madrid, Spain; nuria.m.s@cial.uam-csic.es; 2 Department of Nutritional and Physiological Chemistry, Christian Doppler Laboratory for Bioactive Aroma Compounds, Faculty of Chemistry, University of Vienna, 1090 Vienna, Austria; christina.maria.hochkogler@univie.ac.at (C.M.H.); veronika.somoza@univie.ac.at (V.S.)

**Keywords:** α-glucosidase, biscuits, coffee fibre, fructoologosaccharides, GLP-1, serotonin, stevia, non-nutritive sweeteners

## Abstract

This study assessed the in vitro effects of the bioaccessible food components released during the simulated human digestion of a coffee fibre-containing biscuit (CFB) on α-glucosidase activity, antioxidant capacity and satiety hormones. Digest of CFB presented a significantly (*p* < 0.05) lower amount of sugar (68.6 mg/g) and a higher antioxidant capacity (15.1 mg chlorogenic acid eq./g) than that of a sucrose-containing biscuit (SCB). The CFB significantly reduced (*p* < 0.05) α-glucosidase activity (IC50 = 3.3 mg/mL) compared to the SCB (IC50 = 6.2 mg/mL). Serotonin and glucagon-like peptide-1 (GLP-1) release by differentiated Caco-2 and HuTu-80 cells, respectively, was stimulated by the CFB (355% at a concentration of 0.5 mg/mL and 278% at a concentration of 0.05 mg/mL) to the same order of magnitude as those of the SCB. To summarize, the CFB was demonstrated to reduce monosaccharide bioaccessibility, to inhibit a diabetes-related digestive enzyme, and to improve the release of satiety hormones.

## 1. Introduction

The consumption of energy dense foods high in fat and sugar is associated with the prevalence of obesity, type 2 diabetes, cardiovascular diseases and several cancers [1]. The World Health Organization recommends adults and children reduce their daily intake to less than 10% of their total energy intake. A further reduction to below 5% or roughly 25 g (6 teaspoons) per day would provide additional health benefits [2]. Increasing the price of unhealthy foods through sugar taxes could potentially discourage the overconsumption of sugar [1]. However, multiple strategies are needed to battle obesity and associated comorbidities, e.g., diabetes.

Foods containing both low-sugar and high-fibre can help regulate blood glucose levels, improve satiety, and control body weight gain [3]. Recently, high-fibre and sugar-free foods containing stevia as a non-nutritive sweetener have been developed by our group [4]. Stevia glycosides have been shown to optimize blood sugar and insulin levels in diabetics [5]. Foods high in fibre, such as galactomannan, have been demonstrated to delay gastric emptying, and to inhibit diabetes-related digestive enzymes [6,7], which may influence the feeling of fullness and enhance postprandial hyperglycaemia. Dietary fibre is known to play a key role in the management of obesity and type 2 diabetes [8,9].

The α-glucosidase is a membrane-bound intestinal enzyme, essential for degrading oligosaccharides to monosaccharides [10]. Food constituents such as dietary fibre, stevia and coffee phenols have been shown to act as glucosidase inhibitors [5,7,11], and to reduce postprandial hyperglycaemia. However, results on these compounds present in complex food matrices are missing. 

Intake of satiety-inducing foods becomes a strategy to reduce sugar intake and related chronic diseases. Food intake activates the secretion of several gut-derived mediators [12], including the hormones localized in intestinal enterochromaffin cells, serotonin and glucagon-like peptide-1 (GLP-1). GLP-1 plays a role in the regulation of food intake and presents glucometabolic effects, and serotonin is implicated in the control of satiety [12,13]. 

The aim of this study was to elucidate whether a replacement of sucrose by stevia, coffee fibre and fructooligosaccharides (FOS) in a sucrose-containing biscuit (SCB) improves its antioxidant capacity and the release of gut satiety hormones in vitro, as well as the effect on α-glucosidase activity. We report novel in vitro data on the bioaccesibility of the bioactive compounds of a coffee fibre-containing biscuit (CFB). This study also provides new evidence for using coffee fibre as a sustainable and functional food ingredient.

## 2. Materials and Methods 

### 2.1. Chemicals

Bradford reagent was provided by Bio-Rad Laboratories S.A (Munich, Germany). The following chemicals were obtained from Sigma-Aldrich (St. Louis, MO, USA): α-amylase from human saliva (type IX-A), porcine pepsin from gastric mucosa (3.200–4.500 U/mg protein), pancreatin from porcine pancreas, porcine bile extract, bovine serum albumin (BSA), chlorogenic acid (CGA) (3-CGA), 2,2′-azino-bis (3-ethylbenzothiazoline-6-sulphonic acid (ABTS)), 6-hydroxy-2,5,7,8-tetramethylchroman-2-carboxylic acid (Trolox), phenol 5% (*w/v*), potassium persulphate, Folin-Ciocalteu reagent, *N*α-Acetyl-l-lysine, ortho-phthalaldehyde (OPA), α-glucosidase from intestinal acetone powders of rat, 4-methylumbelliferyl α-d-glucopyranoside(4-MUG), acarbose, 3-(4,5-dimethyl-2-thiazolyl)-2,5-diphenyltetrazolium bromide (MTT) and trypan blue solution. We used the d-mannose/d-fructose/d-glucose assay kit and galactomannan assay kit from Megazyme International Ireland Ldt. (Bray, Ireland), Multi Species GLP-1 Total ELISA kit (Cat-n°. EZGLP1T-36K) from EMD Millipore (St. Charles, MO, USA) and Serotonin High Sensitive ELISA kit (Cat-n°. EA630/96) from DLD Diagnostika GmbH (Hamburg, Germany). For cell culture, Dulbecco’s modified Eagle’s medium (DMEM), minimum essential medium Eagle (MEM), l-glutamine, penicillin and streptomycin were purchased from Sigma-Aldrich (St. Louis, MO, USA). Fetal bovine serum (FBS) was from GIBCO Invitrogen (Karlsruhe, Germany). Water was purified using the Milli-Q and Elix system. All other chemicals and reagents were of analytical grade.

### 2.2. Apparatus and Materials

BioTek powerWaveTM XS (BioTek Instruments, (Winooski, VT, USA), FP-6200 (JASCO, Easton, MD, USA) and Infinite^®^ 200 PRO multimode (TECAN, Deutschland GmbH, Crailsheim, Germany) microplates readers, convection oven (Romag S.A, Barcelona, Spain), UN 500 universal oven (Memmert, Schwabach, Germany) and Telstar Lyobeta-15 lyophilizer (Telstar, Madrid, Spain) were used for analyses. Forcell culture assays, a Neubauer cell counting chamber (0.100 mm depth, 0.0025 mm^2^) (Paul Marienfeld GmbH & Co., KG, Lauda-Königshofe, Germany), an incubator (BINDER GmbH, Tuttlingen, Germany), a Thermo Scientific™ MSC-Advantage™ class II biological safety cabinet (Thermo Fisher Scientific, Waltham, MA, USA), an autoclave (3870EA, Tuttnauer, Hauppauge, NY, USA), a TR400-SW TRINO microscope (VWR, Vienna, Austria), CELLSTAR^®^ multiwell culture plates and standard cell culture flasks (Greiner Bio-One GmbH, Kremsmuenster, Austria) were used.

### 2.3. Food Ingredients

Spent coffee grounds (SCG) from Robusta instant coffee were used as a source of antioxidant coffee fibre [4]. SCG were supplied by Prosol S.A., Palencia, Spain) and stored at −20 °C until use. FOS powder (ORAFTI^®^P95) was from Beneo-Orafti, Barcelona, Spain) and stevia sweetener powder, containing 3% steviol glycosides and 97% maltodextrin, was purchased at a local supermarket. All other basic ingredients were purchased at specialized and certified food markets.

### 2.4. Food Samples

Biscuit formulations are shown in Table 1. Biscuits were prepared following the recipe described by Martinez-Saez et al. [4]. Briefly, the dough was prepared by mixing salt, baking powder and sucrose or stevia. Mineral water was added to the dry mixture and thoroughly blended. In a separate bowl, lecithin and oil were mixed and then added to the mixture. Finally, flour, coffee fibre and FOS were gradually added and the dough was kneaded. The dough was set for 30 min, and shaped into discs. Biscuits were baked at 185 °C for 16 min in an air recirculation oven. Two sets of three biscuits were baked in duplication (*n* = 6). Biscuits were placed in the centre of the tray in order to reduce process variability during baking. Then, biscuits were ground to obtain a representative sample for further analyses. 

Biscuits were digested in triplicate under in vitro oral gastrointestinal human digestion conditions [4]. Briefly, all three stages, salivary (pH 6.9, 10 mL, 5 min, 3.9 U α-amylase/mL, aerobic), gastric (pH 2, 13 mL, 90 min, 71.2 U pepsin/mL, aerobic), and abiotic duodenal step (pH 7, 16 mL, 150 min, 9.2 mg pancreatin and 55.2 mg bile extract/mL, aerobic), were performed in the same flask. Digestion was then centrifuged and the soluble fractions containing bioaccessible compounds, able of being absorbed and metabolized, were treated with cholestyramine resin (10% *w/v*) to remove the bile acids and mimic the human bile salt reabsorption. Then, soluble fractions were frozen at −20 °C, lyophilized and stored at room temperature until further analysis.

Coffee fibre, stevia and FOS were also digested in vitro following the procedure described above to gain insight into their contribution to bioactive compounds. 

### 2.5. Cell Culture: Caco-2 and HuTu-80

The human colon cancer cell line (Caco-2) was cultured in DMEM supplemented with FBS (10%), l-glutamine (2%) and penicillin/streptomycin (1%) at 37 °C and 5% CO_2_. Cells were grown in 12-well plates until reaching confluence, after approximately 3 days. Cell differentiation was obtained by subsequent culture for 21 days including medium changes every two to three days. The enterocyte-like differentiated Caco-2 cells were then used for further studies on serotonin hormone release. 

The human duodenal cancer cell line (HuTu-80) was cultured in MEM supplemented with FBS (10%), l-glutamine (2%) and penicillin/streptomycin (1%) at 37 °C and 5% CO_2_. Cells were grown in 24-well plates until reaching confluence, after approximately 1 day. HuTu-80 cells were not differentiated with enterocyte-like properties, and 24 h after seeding, they were ready to be used for analyses on GLP-1 hormone release.

After thawing, both cells lines were passaged three to four times to give cells time to recover to their normal growth rate.

### 2.6. Bioaccessibility of Food Components

Digests of coffee fibre, stevia and biscuits (SCB and CFB) were characterized.

#### 2.6.1. Total Carbohydrates

Total carbohydrates were determined using the phenol–sulphuric method as described by Masuko et al. with slight modifications [14]. Samples (20 μL) were mixed with concentrated sulphuric acid (93–98%) (61 μL) and phenol solution (5%, *w/v*) (18 μL) in a multi-well plate. After incubation at 90 °C for 5 min in a water bath, the microplate was cooled to room temperature and absorbance was measured at 490 nm. The calibration curve was constructed using glucose (0.1–0.9 mg/mL) as a standard. Reagent blank and sample blank were also prepared and analysed in each set of samples. All measurements were performed in triplicate and results were expressed as mg glucose equivalents (eq.)/g digest.

#### 2.6.2. Galactomannan

Galactomannan content determination was performed using an enzymatic kit following the manufacturer’s instructions. The method was adapted to a micromethod format. Analyses were carried out in triplicate and results were expressed as mg/g digest.

#### 2.6.3. Free Sugars 

Glucose, fructose and mannose contents were determined using an enzymatic kit following the manufacturer’s instructions. The method was adapted to a micromethod format. The analysis was performed in triplicate. Results were expressed as mg glucose, mg fructose and mg mannose/g digest.

#### 2.6.4. Soluble Proteins and Peptides

The Bio-Rad Protein Assay, based on the Bradford method in a micromethod format, was used to determine proteins and peptides. Reagents were prepared according to the manufacturer’s instructions. Briefly, a solution of Bradford reagent (1:4 reagent:milli-Q water) was prepared and filtered. Ten μL of sample and 200 μL of Bradford solution were placed in a multi-well plate. After 5 min of incubation at room temperature, absorbance was measured at 595 nm. Sample blank and reagent blank were also analysed. BSA was used as a standard (0.05–1 mg/mL). All measurements were performed in triplicate. Results were expressed as mg BSA eq./g digest. 

#### 2.6.5. Free Amino Groups

The release of amino acids by the proteolysis of proteins forming wheat flour and coffee fibre was measured by the OPA assay according to Go et al. [15]. OPA reagent was freshly prepared by dissolving 10 mg OPA in 250 μL ethanol (95%, *v/v*), 9.8 mL phosphate buffered saline solution PBS (10 mM, pH 7.4) and 20 μL β-mercaptoethanol. The reaction was carried out in a 96-well microtest plate by mixing 10 μL sample, 140 μL PBS and 100 μL OPA reagent. Fluorescence was read at 360 ± 40 nm excitation and 460 ± 40 nm emission wavelengths for 15 min at 37 °C. The calibration curve was constructed using standard solutions of *N*α-acetyl-l-lysine (0.025–1 mM). All measurements were performed in triplicate, and data were expressed as mg *N*α-acetyl-l-lysine eq./g digest.

These two methods (2.6.4. and 2.6.5.) provide complementary data regarding the compounds released during in vitro hydrolysis of the macronutrient (protein) during the digestive process.

#### 2.6.6. Total Phenolic Content 

Total phenolic content (TPC) was determined by the Folin–Ciocalteu method as described by Contini et al. [16], adapted to a micromethod format. Ten μL of sample and 150 μL of Folin–Ciocalteu solution were incubated at room temperature for 3 min and 50 μL of sodium bicarbonate were added. Reaction was run for 120 min at 37 °C, and absorbance was read at 735 nm. Sample blank and reagent blank were also analysed in each set of samples. The CGA calibration curve (0.1–1 mg/mL) was used for quantification. Measurements were performed in triplicate and results were expressed as mg CGA eq./g digest.

#### 2.6.7. Antioxidant Capacity

The overall antioxidant capacity of the digested biscuits and coffee fibre was analysed using the indirect ABTS^•+^ decolourisation assay as described by Oki et al. [17]. An ABTS^•+^ stock solution was prepared by adding 140 mmol/L potassium persulfate (44 μL) to a 7 mmol/L ABTS^•+^ aqueous solution (2.5 mL), and the mixture was then left to stand for 16 h at room temperature. The working solution of the radical ABTS^•+^ was prepared by diluting the stock solution 1:75 (*v/v*) in a sodium phosphate buffer (5 mmol/L, pH 7.4) to obtain an absorbance value of 0.7 ± 0.02 at 734 nm. Samples (30 μL) were added to ABTS^•+^ solution (270 μL) in a microplate. Absorbance was measured at 734 nm for 10 min at 30 °C. CGA (0.025–0.25 mmol/L) and trolox (0.025–0.25 mmol/L) were used for quantification. All measurements were performed in triplicate and results were expressed as mg CGA eq./g digest.

### 2.7. Health-Promoting Properties of Bioaccessible Food Components

#### 2.7.1. Alpha-Glucosidase Inhibition Assay

The α-glucosidase inhibitory activity of the digested biscuits, coffee fibre, stevia and FOS was analysed following the methodology described by Berthelot et al. and Geddes et al. [18,19] with slight modifications. An alpha-glucosidase enzyme was extracted previous to the assay. Briefly, 100 mg of rat intestine powder were dissolved in 3 mL NaCl (0.9%), sonicated in an ice bath for 6 min and then centrifuged at 10,000 g for 30 min. The supernatant containing the enzyme was stored in the freezer. In a 96-well microplate, 100 μL of sample dissolved in PBS (100 mM, pH 6.9) were mixed with 100 μL α-glucosidase (diluted 1/10) and 100 μL 4-MUG (2 mM). Fluorescence was then monitored at an excitation wavelength of 360 nm and an emission wavelength of 460 nm for 30 min at 37 °C. Blank of sample and negative controls (buffer, enzyme and 4-MUG) were included. Acarbose was used as a positive control (standard inhibitor). Curves of samples and acarbose were assayed to cover the whole range of inhibition of the enzyme (~0.5–96%). The percentage (%) of α-glucosidase inhibition was calculated using the equation:

(1) $$\alpha -\mathrm{glucosidase inhibition}\;(\%)\;=\;\frac{F_{\mathrm{nc}}{-F}_{s}}{F_{\mathrm{nc}}}\times 100$$

where $F_{\mathrm{nc}}$ is the fluorescence of the negative control (without inhibitor) and $F_{s}$ is the fluorescence of the sample. All measurements were performed in triplicate. Results were expressed as the concentration causing 50% inhibition (IC50 mg/mL).

#### 2.7.2. Assays of Serotonin and GLP-1 Secretion

Soluble fractions recovered from the digested SCB, CFB and coffee fibre containing bioaccessible compounds for absorption and metabolism were used to stimulate the secretion of satiating hormones in cell cultures. The effect of stevia on the release of satiating hormones was also tested.

- Cell viability: Cytotoxic effects of test samples were excluded by performing the colorimetric MTT assay [20]. Differentiated Caco-2 cells were incubated with samples (0.05, 0.5 and 5 mg/mL) diluted in PBS containing ascorbic acid (0.1%) and after a 5-min exposure to cells, samples were removed. HuTu-80 cells were first starved with a serum free medium, glucose and glutamine, for 1 h prior to the incubation with the samples (0.01, 0.05 and 0.5 mg/mL) diluted in the starving-medium. After 90-min of exposure, samples were finally removed from HuTu-80 cells.

In both cellular lines, exposure was carried out at 37 °C and a negative (medium) and positive control (DMSO) were also included. The MTT solution (50 mg/mL, 1:6) was incubated for 10–15 min and the resulting formazan diluted in DMSO was measured at 570 nm. Viability was determined relative to untreated negative control cells (100%). Three groups of different passaging and samples in duplicate were performed in each set of analysis (*n* = 3, tr = 6). The percentage of cell vitality was calculated as follows: vitality (%) = (dead cell number/total cell number) × 100. Results were expressed as total cell viability/control (T/C) [%].

- Stimulation and quantification of serotonin: Caco-2 cells were supplemented with the bioaccessible fractions (150 μL) at three different concentrations (0.5, 0.05, 0.01 mg/mL) in duplicate. Cells were washed with PBS prior to the addition of samples. Then, cells were stimulated for 5 min in darkness in an orbital shaker. Supernatants were removed from cells and frozen until further quantification of the serotonin hormone could be completed. Positive (cinnamaldehyde, 5 mM) and negative (buffer) controls were also tested.

The serotonin released by Caco-2 cells was quantified in darkness using a highly sensitive enzyme immunoassay kit (ELISA competitive) following the manufacturer’s instructions. Three groups of different passage numbers were performed in each set of analysis (*n* = 3, tr = 6). Results were expressed as T/C [%] compared to the control.

- Stimulation and quantification of GLP-1: HuTu-80 cells were first starved with a serum free medium, glucose and glutamine, for 1 h prior to stimulation with the bioaccessible fractions (500 μL) at 0.01, 0.05 and 0.5 mg/mL in duplicate. Positive (glutamine 40 mM) and negative (medium) controls were also tested. Cells were exposed to samples for 90 min at 37 °C. After stimulation, supernatants were collected and frozen until further quantification of the GLP-1 hormone could be completed.

The GLP-1 hormone released from HuTu-80 cells was quantified using the sandwich ELISA kit following the manufacturer’s instructions. Three groups of different passage numbers were performed in each set of analysis (*n* = 3, tr = 6). Results were expressed T/C [%] compared to the control.

### 2.8. Statistical Analysis

Statistical analyses were performed using SigmaPlot 11.0 (Systat Software Inc., San Jose, CA, USA). Data were expressed as the mean value ± standard deviation for all analyses except for those comprising cell culture which were expressed as mean ± standard error of the mean (SEM). Differences between means were determined through the analysis of variance (ANOVA), one-way ANOVA, followed by Dunn’s, Fisher LSD or Holm–Sidak post-hoc tests. Differences were considered to be significant at *p* < 0.05.

## 3. Results and Discussion

### 3.1. Bioaccessibility of Food Components 

Table 2 shows data on the bioaccessible food components released during the digestion process. In the case of coffee fibre, the major components of the bioaccessible fraction of the digest were carbohydrates (11.3%), whereas only trace amounts of sugars such as glucose (0.01%), fructose (0.02%) and mannose (0.03%) were detected. In contrast, a higher content in polysaccharides like galactomannan (2%) was quantitated. TPC represented 1.56% of the bioaccessible food components, and proteins (0.88%) and free amino groups (2.2%) were also present. These results suggest that SCG used as coffee fibre mainly provides non-digestible, complex carbohydrates to the bioaccessible fraction, which is in accordance with the literature [4,21].

In the case of SCB, the major components of the bioaccessible fraction of the digest were also carbohydrates (64.8%). Sugars represented 18% of total carbohydrates. Fructose (52.3%) and glucose (47%) were found in the highest amounts, followed by trace amounts of mannose (0.7%). Protein and amino acid contents were lower than 1% (0.41% and 0.80%, respectively) and TPC represented 0.85%. Wheat flour starch seems to be the main contributor to the carbohydrate content of the biscuit (Table 1). According to the literature, wheat flour is composed of 65% digestible starch [22], which is hydrolysed by digestive enzymes—salivary and pancreatic α-amylases—to glucose molecules and oligosaccharides [23]. Sucrose added to the traditional formulation can also be converted into glucose and fructose, mainly by chemical reactions that have occurred during the processing of cereal-based products [24], and the acid conditions of the stomach [25], since the intestinal sucrase enzyme was not used in this particular digestion model. These available carbohydrates may turn the traditional biscuit into a food containing high sugar levels, which may consequently cause fast postprandial blood glucose glycaemic responses [26]. High sugar diets are associated with decreased satiety and increased glucose intolerance, a greater risk of overweight and obesity, and impaired lipid metabolism [27]. Furthermore, glucose and fructose follow different metabolic pathways after their absorption, resulting in different effects on blood glucose concentrations. Obesity is related to an elevated intake of both sugars. Type 2 diabetes is associated with high glucose diets, while the overconsumption of fructose is associated with non-alcoholic fatty liver diseases and augmented de-novo triglyceride synthesis [28].

The bioaccesibility of glycaemic sugars in CFB was significantly (*p* < 0.05) lower, with reductions of 46.4% ± 8.8% and 35.6% ± 1.1% for glucose and fructose, respectively, compared to the SCB (Table 2). The stevia sweetener did not significantly contribute to the sugar content of the CFB. Stevia, in particular steviol glycosides, cannot be hydrolysed by digestive enzymes in the small intestine. Stevia is metabolised to steviol by the microbiota of the colon [29]. However, our digestion conditions did not include the effect of the microbiota on the digestibility of the biscuits and their components. Stevia added to the CFB contained maltodextrin 97% as an additive. Maltodextrin composing stevia seems to have a significantly lower contribution to the bioaccesibility and total glycaemic carbohydrates of the food compared to that observed for sucrose in SCB formulation. Sucrose replacement by stevia played an important role in lowering the bioaccesibility of the glycaemic sugars in foods. Moderate glucose levels found in CFB are a necessary primary energy source for proper cell function in the organism [30]. On the other hand, galactomannan was present in the CFB unlike the SCB. Coffee fibre included in the CFB is a natural source of galactomannan (Table 2). Intake of galactomannan is associated with reduced weight gain, adiposity, liver fat and blood glucose levels [31], making coffee fibre an attractive ingredient for confectioneries. Moreover, FOS incorporated in the CFB as soluble fibre has been shown to reduce post-prandial glycaemic responses [32]. Marangoni and Poli [27] also obtained a markedly lower glycaemic index in bread and biscuits by adding a proprietary fibre mixture to their formulations. 

Regarding the antioxidant properties of the biscuits, significant differences (*p* < 0.05) were found between the CFB and the SCB. Bioaccessibility of antioxidants was estimated as the overall antioxidant capacity of food digests.The digestion of the CFB released a significantly greater amount of antioxidants (15.07 ± 1.45 mg CGA eq./g digest) than in the SCB (10.43 ± 0.90 mg CGA eq./g digest). Most of the antioxidants of the SCB may be ascribed to phenolic compounds (Table 2); in contrast, the CFB might also contain other non-phenolic antioxidants which may contribute to its overall antioxidant capacity. The bioaccessible fraction of the digested coffee fibre had a high antioxidant character (46.14 ± 3.61 mg CGA eq./g digest). Furthermore, stevia [33], FOS [34], and gluten peptides released during the digestion process by chemical and enzymatic hydrolysis [35] may also exert an antioxidant character. 

The high antioxidant properties of the CFB may play an important role in reducing the risk of obesity and diabetes. Consequently, introducing food antioxidants through the diet may be of great interest. High-antioxidant diets have been related to reduced inflammation and increased circulating antioxidants in cross-sectional and randomized intervention studies [36]. 

In summary, the bioaccessibility of the analysed nutrients, such as total carbohydrates, proteins, amino acids and phenols, was not significantly different (*p* > 0.05) between the two biscuits. However, the carbohydrate profile of CFB was enhanced by replacing sucrose with stevia, FOS and coffee fibre, providing the biscuits with the potential to augment satiety and reduce hyperglycaemia [26].

### 3.2. Health-Promoting Properties of Foods

#### 3.2.1. Inhibition of α-Glucosidase Activity

**Figure 1:**
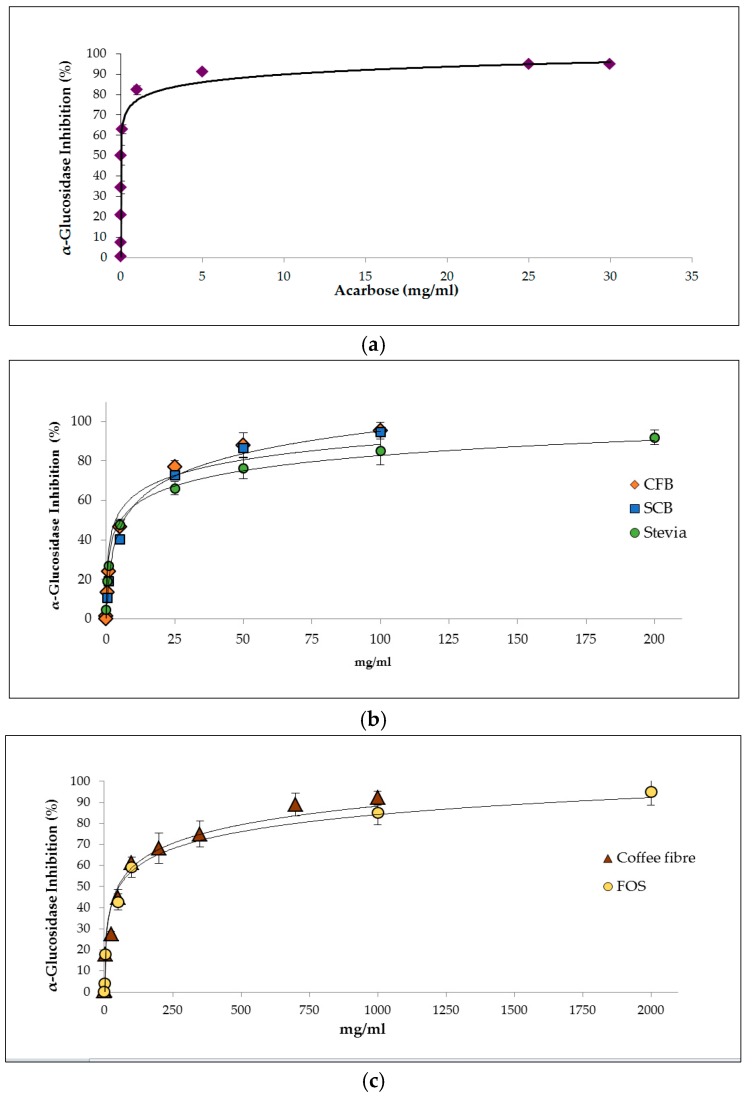
Effect on α-glucosidase activity is represented by dose-response curves of (**a**) acarbose (0.1 μg/mL–30 mg/mL); (**b**) soluble fractions recovered from the digested sucrose-containing biscuit (SCB) (0.5–100 mg/mL), coffee fibre-containing biscuit (CFB) (0.01–100 mg/mL) and stevia (0.05–200 mg/mL); and (**c**) soluble fractions recovered from the digested coffee fibre (0.5–1000 mg/mL) and FOS (0.5–2000 mg/mL). Values represent mean ± standard deviation. This includes a duplicate of sample preparation and a triplicate of analysis (*n* = 6).

IC50 values for α-glucosidase inhibition were calculated from dose–response curves (Figure 1a–c). The IC50 for acarbose was 4.4 μg/mL. Alpha-glucosidase inhibitors were detected in the digests of the samples. However, the content of α-glucosidase inhibitors released during digestion differed significantly (*p* < 0.05) between samples. The CFB presented the lowest IC50 value of all the studied samples (Table 3). According to the literature, the CFB is a better inhibitor than other food products such as lemon (IC50 36.59 mg/mL), lime (10.96 mg/mL), grapefruit (62.10 mg/mL) [37], green tea (11.1 mg/mL), sardine muscle hydrolysate (48.7 mg/mL) and yogurt (519.8 mg/mL) [38].

Stevia, FOS and coffee fibre are demonstrated to contribute to the inhibitory effect of the α-glucosidase observed for the CFB, although stevia had a higher inhibitory capacity than the other two ingredients. The inhibitory effect of stevia against α-glucosidase has been previously described [11]. Phenolic compounds released during coffee fibre digestion may also act as α-glucosidase inhibitors; for instance, the main phenolic compound of coffee, CGA, could have potential benefits on type 2 diabetes [39]. Other compounds released during the digestion of the CFB such as bioactive peptides could also exhibit anti-diabetic properties [40]. Proof-of-principle human intervention studies are needed to verify this effect.

#### 3.2.2. Release of Satiety Hormones

Different doses of the tested samples were not cytotoxic (Figure S1, Supplementary Material).

**Figure 2:**
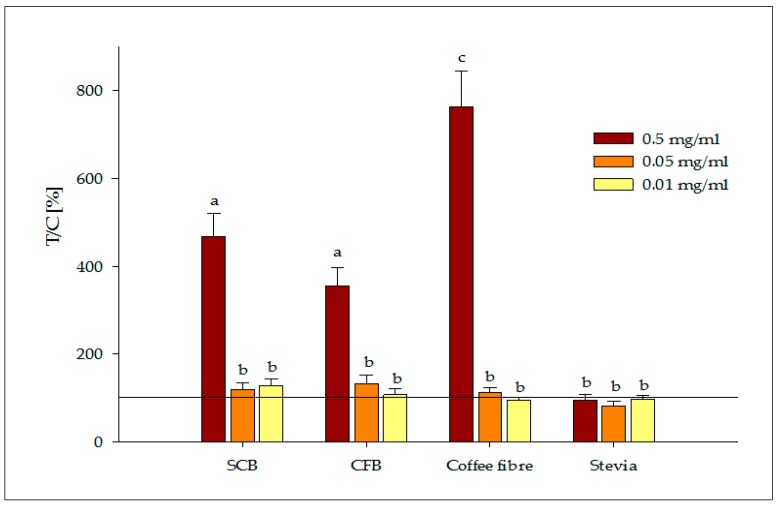
Serotonin release after stimulation of Caco-2 cells at 0.5, 0.05 and 0.01 mg/mL with soluble fractions recovered from the digested sucrose-containing biscuit (SCB), coffee fibre-containing biscuit (CFB) and antioxidant coffee fibre that contain bioaccessible compounds, as well as stevia. Results are displayed as T/C in percent compared to the control (cells with media = 100%). All measurements were expressed as mean ± SEM (*n* = 3, tr = 6). Significant differences between treatments were tested with one-way ANOVA followed by the Holm–Sidak post-hoc test (*p* < 0.05) and marked with the letters a, b and c.

The samples’ effect on cellular secretion of serotonin is shown in Figure 2. Caco-2 cells treated with 0.5 mg/mL of the CFB digest exhibited a four-fold increase in serotonin secretion (355%). No differences in serotonin release were found (*p* > 0.05) between the digests of the SCB and the CFB. However, compounds released during the digestion of the antioxidant coffee fibre had a significant effect on the secretion of serotonin, increasing serotonin release seven-fold (763%) compared to the basal level. To the best of our knowledge, this is the first time that the effect of the antioxidant coffee fibre on satiety hormones has been reported. Gostner et al. found that coffee compounds such as gallic acid and caffeic acid had the potential to increase tryptophan availability, needed for the biosynthesis of serotonin, via inhibition of indoleamine 2,3-dioxygenase, which is involved in tryptophan metabolism [41]. Further studies are required to elucidate which of the compounds released during the digestion of coffee fibre is responsible for its satiating effect. 

In contrast, the natural non-nutritive sweetener, stevia, did not stimulate serotonin secretion in differentiated Caco-2 cells at the tested concentrations. This result is in agreement with that described by Ripken et al., which did not find serotonin release from porcine intestinal tissue segments when incubated with Rebaudioside A, the sweetest glycoside of stevia [42]. Moreover, Anton et al. reported in human intervention studies that the intake of preloads containing stevia had satiety levels similar to those of sucrose preloads, measured by the subjective visual analogue scale [43]. Therefore, more investigations are required to clarify the role of this hypocaloric sweetener and its metabolites in serotonin secretion. 

Other bioaccessible compounds may exhibit serotonin stimulation. Gluten peptides from wheat flour have also been shown to contribute to higher hypothalamic and cortical serotonin levels in animal models [44]. Phenolic compounds can also play an important role in appetite suppression by stimulating this important signalling molecule [45]. Furthermore, Maillard reaction products such as N(ε)-carboxymethyl lysine from heat-treated foods like biscuits, have been found to contribute to satiety regulation through central/brain serotonin release in SH-SY5Y cells [46]. Therefore, biscuits made using sucrose as a sweetener might contain these advanced products of the Maillard reaction that could contribute to the stimulation of serotonin. However, replacing sucrose with stevia limits the progress of Maillard reaction in food toward advanced stages [47], and may not significantly contribute to a postprandial satiating effect of the food prepared according to the CFB. Despite the controversial effect of peripheral serotonin on satiety [48], there is evidence that peripheral serotonin has similar effects on satiety as central serotonin does [13,49,50]. Further studies are needed to identify the individual food components that contribute to the release of serotonin from the gut in the CFB and the SCB.

**Figure 3:**
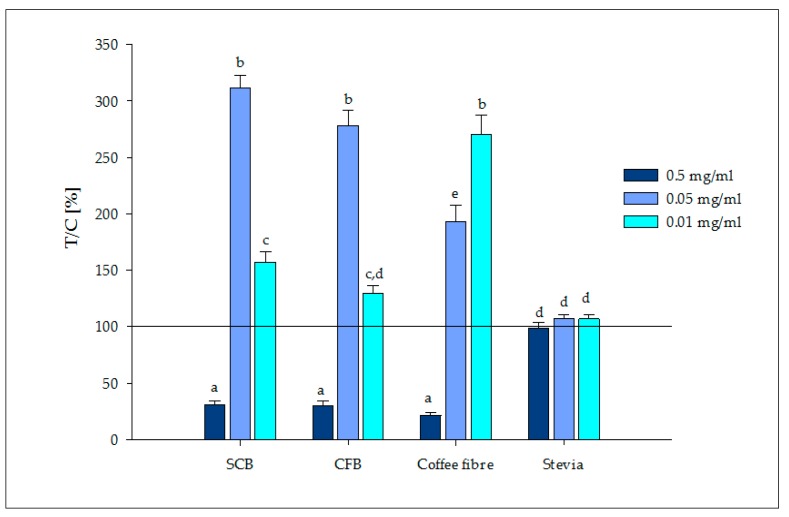
Glucagon-like peptide-1 (GLP-1) release after stimulation of HuTu-80 cells at 0.5, 0.05 and 0.01 mg/mL soluble fractions recovered from the digested sucrose containing biscuit (SCB), coffee fibre containing biscuit (CFB) and antioxidant coffee fibre that contain bioaccessible compounds, as well as stevia. Results are displayed as T/C in percent compared to control (cells with media = 100%). All measurements were expressed as mean ± SEM (*n* = 3, tr = 6). Significant differences between treatments were tested with one-way ANOVA followed by the Holm–Sidak post-hoc test (*p* < 0.05) and marked with the letters a, b, c, d and e.

Regarding cellular secretion of GLP-1, significant GLP-1 values were obtained (*p* < 0.05) for the stimulation of HuTu-80 cells with the bioaccessible compounds released during the digestion of food formulations (Figure 3). Compared to non-treated control cells, GLP-1 release was significantly higher (*p* < 0.05), i.e., three times higher (278%), after incubation of HuTu-80 cells with the soluble fraction of the digested novel CFB (0.05 mg/mL). No significant differences were found (*p* > 0.05) between the SCB and the CFB. The same trend was observed for all concentrations of the biscuits. 

Compounds released during the digestion of antioxidant coffee fibre from SCG significantly stimulated the secretion of the GLP-1 hormone. Although researchers have previously reported GLP-1 stimulation caused by coffee beverages in cells, mice and humans [51], this is the first report of the effect of coffee fibre from SCG. 

On the other hand, stevia was not effective at the three tested concentrations, which is in agreement with the results of Fujita et al. for an in vivo animal model [52]. FOS present in the CFB (Table 1) have been associated with appetite suppression via stimulation of GLP-1 release [53]. Other bioaccessible compounds, such as proteins and peptides, may also stimulate GLP-1 release. [54]. In addition, intact and digested wheat proteins have been found to produce satiety and have an anti-diabetic effect through GLP-1 stimulation [55]. Further studies are required to characterize food compounds released during digestion and to determine those that contribute to the satiating effect of the biscuits.

## 4. Conclusions

To the best of our knowledge, this is the first report of an inhibitory effect on α-glucosidase activity and release of gut satiety hormones (GLP1 and serotonin) by bioaccessible compounds produced during the digestion of a CFB and its ingredients, antioxidant coffee fibre, FOS and stevia. Changes in the traditional biscuit formulation significantly (*p* < 0.05) reduced monosaccharide bioaccesibility and added novel properties to the biscuits. In addition, we provided new evidence of the use of antioxidant coffee fibre as a sustainable and functional food ingredient. Compounds released during digestion of coffee fibre affected the secretion of serotonin and GLP-1. Stevia resulted better inhibitor of α-glucosidase than FOS and coffee fibre. However, stevia was infective on the secretion of hormones. Results derived from this research suggest the use of this particular dietary fibre in food formulations in concentrations corresponding to nutritional claims “source of fibre” (≥3%) and “high fibre” (≥6%) might also provide bioactive compounds at physiological concentrations which might be able to exert a satiety effect. Proof-of-concept human intervention studies must verify whether the combination of coffee fibre, FOS and stevia may improve the sensorial quality and health promoting properties of the food, satisfying consumers’ demands.

## Figures and Tables

**Table 1:** Biscuit formulations: sucrose-containing biscuit (SCB) and coffee fibre-containing biscuit (CFB) made with stevia, coffee fibre and fructooligosaccharides (FOS).

Ingredients (g)	SCB	CFB
Wheat flour	56.00	59.40
Water	20.00	21.20
Sunflower oil	7.75	8.20
Baking powder	0.55	0.58
Salt	0.37	0.39
Soy lecithin	0.33	0.35
Sucrose	15.00	-
Stevia	-	2.10
FOS	-	3.50
Coffee fibre	-	4.20
TOTAL dough	100	100
Estimated fibre content (g fibre/100 g biscuit)	1.90	7.50 ^1^

^1^ “High fibre content” (≥6 g fibre/100 g).

**Table 2:** Bioaccessible compounds released during in vitro oral-gastrointestinal digestion of a sucrose-containing biscuit (SCB), a coffee fibre-containing biscuit (CFB), coffee fibre and stevia.

Bioaccessible Compounds	SCB	CFB	Coffee Fibre	Stevia
Total Carbohydrates				
mg glucose eq./g digest	647.01 ± 70.0 ^a^	609.34 ± 17.20 ^a^	113.66 ± 7.57 ^b^	n.d.
Galactomannan				
mg/g digest	ND	1.60 ± 0.11 ^a^	19.49 ± 1.19 ^b^	n.d.
Sugars				
mg glucose/g digest	54.84 ± 9.94 ^a^	28.78 ± 0.65 ^b^	0.01 ± 0.00 ^c^	40.02 ± 0.57 ^d^
mg fructose/g digest	60.91 ± 3.31 ^a^	39.21 ± 1.48 ^b^	0.21 ± 0.01 ^c^	6.20 ± 0.46 ^d^
mg mannose/g digest	0.74 ± 0.11 ^a^	0.59 ± 0.11 ^a^	0.29 ± 0.01 ^b^	ND
Soluble proteins				
mg BSA eq./g digest	4.11 ± 0.09 ^a^	3.88 ± 0.30 ^a^	8.75 ± 0.28 ^b^	n.d.
Free amino groups				
mg *N*α-acetyl-l-lysine eq./g digest	7.95 ± 0.19 ^a^	9.16 ± 0.83 ^a^	24.0 ± 2.53 ^b^	n.d.
Total phenolic content				
mg CGA eq./g digest	8.86 ± 0.49 ^a^	8.98 ± 0.52 ^a^	15.56 ± 0.95 ^b^	n.d.

ND Not Detected; n.d. not determined. Data are presented as mean ± standard deviation. Triplicate of sample preparation and triplicate of analysis (*n* = 9). Different letters indicate significant differences (*p* < 0.05) between the samples of the same row.

**Table 3:** IC50 values are demonstrated against α-glucosidase activity and equivalents of acarbose of the bioaccessible fractions of digested sucrose-containing biscuit (SCB), coffee fibre-containing biscuit (CFB), coffee fibre, stevia and fructooligosaccharides (FOS).

Samples	IC50 (mg/mL)	mg Acarbose eq./g Digest
Acarbose	0.004 ± 0.00 ^a^	-
Stevia	5.53 ± 0.35 ^b,c^	0.79 ± 0.05
SCB	6.22 ± 0.33 ^b^	0.70 ± 0.04
CFB	3.32 ± 0.12 ^c^	1.32 ± 0.05
Coffee fibre	23.9 ± 1.31 ^d^	0.18 ± 0.01
FOS	53.4 ± 2.22 ^e^	0.08 ± 0.00

Values represent mean ± standard deviation (*n* = 2). Differences were tested with one-way ANOVA followed by a Fisher post-hoc test (*p* < 0.05) and marked with letters ^a^, ^b^, ^c^, ^d^ and ^e^.

## Supplementary Materials

The following are available online at www.mdpi.com/2072-6643/9/7/694/s1, Figure S1: Cytotoxic effects of soluble fractions recovered from the digested sucrose-containing biscuit (SCB), coffee fibre-containing biscuit (CFB) and antioxidant coffee fibre that contain bioaccessible compounds; as well as, stevia, on (**a**) Caco-2 cells at concentrations of 5, 0.5 and 0.05 mg/mL; and on (**b**) HuTu-80 cells at 0.5, 0.05 and 0.01 mg/mL, compared to control (cells with media, 100% viability). All measurements were expressed as mean ± SEM (*n* = 3, tr = 6). Significant differences vs. control were determined by One-Way ANOVA followed by Dunn’s post-hoc test (*p* < 0.05) and marked as “*”.
