# Supplementary material for: Biscuits with No Added Sugar Containing Stevia, Coffee Fibre and Fructooligosaccharides Modifies α-Glucosidase Activity and the Release of GLP-1 from HuTu-80 Cells and Serotonin from Caco-2 Cells after In Vitro Digestion
Source: Nutrients. 2017 Jul 4;9(7):694. doi: 10.3390/nu9070694 (PMC5537809; doi:10.3390/nu9070694)
Supplement: Supplementary file 1 [file nutrients-09-00694-s001.pdf]

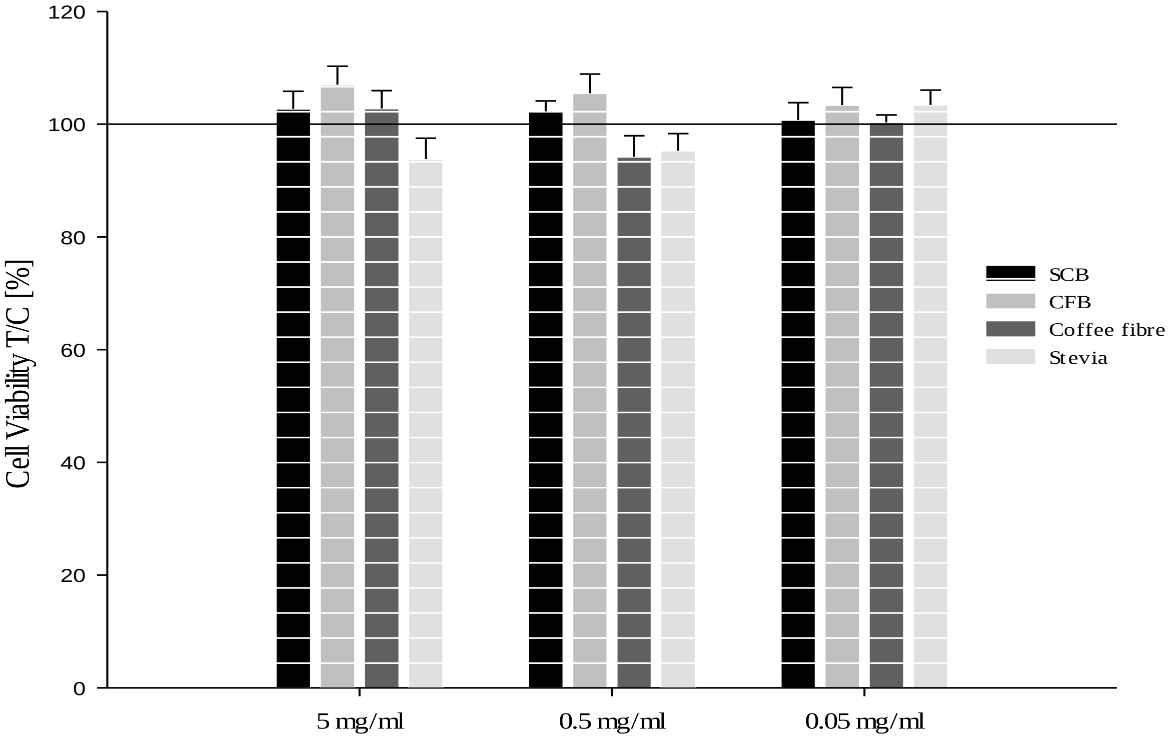

(**a**)

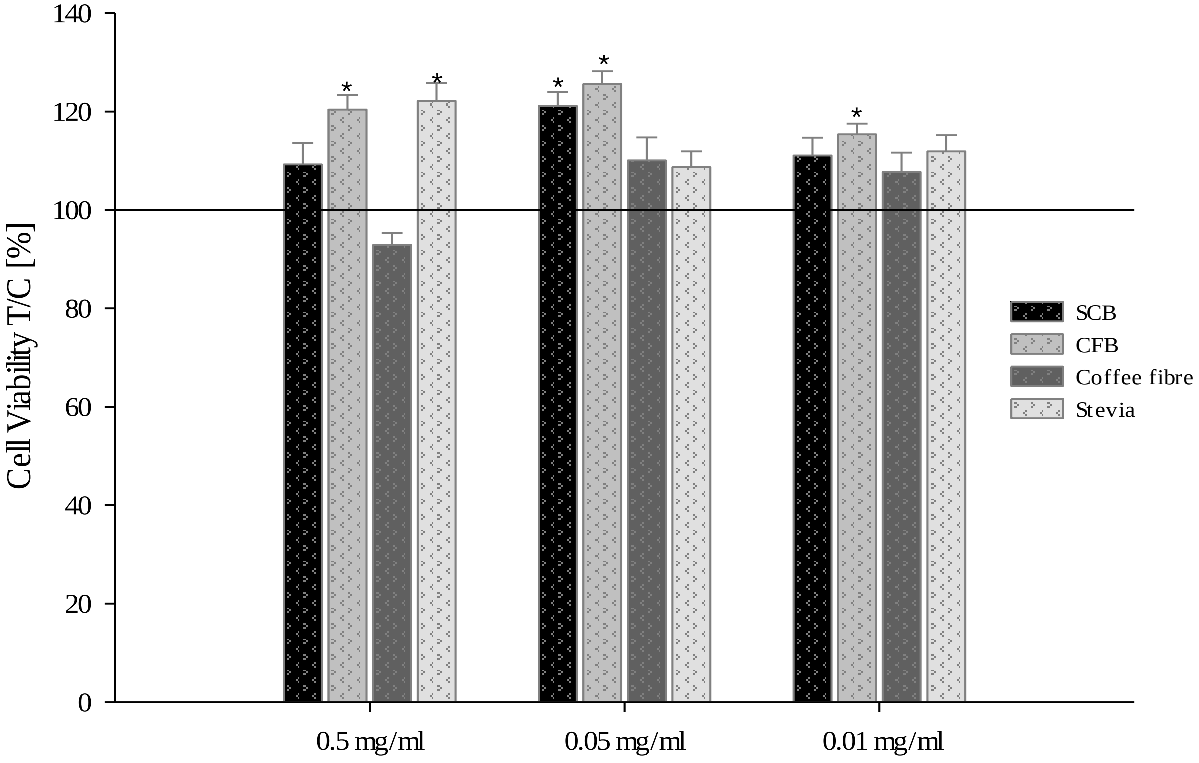

(**b**)

Figure S1. Cytotoxic effects of soluble fractions recovered from the digested sucrose-containing biscuit (SCB), coffee fibre-containing biscuit (CFB) and antioxidant coffee fibre that contain bioaccessible compounds; as well as, stevia, on (**a**) Caco-2 cells at concentrations of 5, 0.5 and 0.05 mg/ml, and on (**b**) HuTu-80 cells at 0.5, 0.05 and 0.01 mg/ml, compared to control (cells with media, 100% viability). All measurements were expressed as mean ± SEM (n=3, tr=6). Significant differences vs. control were determined by One-Way ANOVA followed by Dunn's posthoc test ($p < 0.05$) and marked as '*'.
